# Efficient protocol for the differentiation of kidney podocytes from induced pluripotent stem cells, involving the inhibition of mTOR

**DOI:** 10.1038/s41598-023-47087-8

**Published:** 2023-11-16

**Authors:** Masahiro Yasuda, Tadashi Kato, Mai Okano, Hiromi Yamashita, Yoshikazu Matsuoka, Yasumasa Shirouzu, Tatsuya Fujioka, Fumiyuki Hattori, Shoji Tsuji, Kazunari Kaneko, Hirofumi Hitomi

**Affiliations:** 1 Department of iPS Stem Cell Regenerative Medicine, Kansai Medical University, 2-5-1 Shin-Machi, Hirakata, Osaka 573-1010 Japan; 2 Department of Pediatrics, Kansai Medical University, Osaka, Japan; 3 Division of Nephrology, Department of Medicine, Showa University School of Medicine, Tokyo, Japan

**Keywords:** Pluripotent stem cells, Glomerulus, Podocytes

## Abstract

The mechanistic/mammalian target of rapamycin (mTOR) is involved in a wide range of cellular processes. However, the role of mTOR in podocytes remains unclear. In this study, we aimed to clarify the role of mTOR in podocyte differentiation from human induced pluripotent stem cells (hiPSCs) and to establish an efficient differentiation protocol for human podocytes. We generated podocytes from hiPSCs by modifying protocol. The expression of the podocyte-specific slit membrane components nephrin and podocin was measured using PCR, western blotting, flow cytometry, and immunostaining; and the role of mTOR was evaluated using inhibitors of the mTOR pathway. Nephrin and podocin were found to be expressed in cells differentiated from hiPSCs, and their expression was increased by mTOR inhibitor treatment. S6, a downstream component of the mTOR pathway, was also found to be involved in podocyte differentiation. we evaluated its permeability to albumin, urea, and electrolytes. The induced podocytes were permeable to the small molecules, but only poorly permeable to albumin. We have shown that the mTOR pathway is involved in podocyte differentiation. Our monolayer podocyte differential protocol, using an mTOR inhibitor, provides a novel in vitro model for studies of kidney physiology and pathology.

## Introduction

Podocytes are complex cells that consist of a cell body, major processes, and foot processes, and play important roles in the maintenance of the glomerular filtration barrier and its function^1,2^. Nephrin and podocin are transmembrane glycoproteins produced by podocytes that are responsible for the permselective barrier of the glomerulus^3,4^. The glomerular slit diaphragm facilitates the passage of primary urinary filtrate, but prevents the passage of high-molecular-weight plasma proteins^5^. Podocyte injury or deterioration of glomerular filtration barrier function results in proteinuria, which is a feature of many human kidney diseases, such as nephrotic syndrome, diabetic nephropathy, and congenital diseases. However, the physiological regulation of podocytes and the pathophysiological sequelae to injury have not been well characterized, because the cultivation of human podocytes without immortalization is challenging, and it is difficult to obtain human samples.

Mechanistic/Mammalian target of rapamycin (mTOR), a serine/threonine kinase of the phosphoinositide 3-kinase-related kinase family, is the target of rapamycin in mammals^6^. mTOR senses various environmental and intracellular changes, including in nutrient availability and energy status, and coordinates diverse cellular processes, including cell growth, differentiation, autophagy, survival, and metabolism^7^. It is the core component of two distinct complexes: complex 1 (mTORC1) and complex 2 (mTORC2)^8^, which are respectively sensitive and not sensitive to rapamycin^9^. Several previous studies have shown that podocyte damage involves the mTOR pathway^10,11^. In addition, mTOR inhibition is an effective treatment for both animal and patients with kidney disease^12,13^, but is also associated with the development and exacerbation of proteinuria and glomerulosclerosis^14^. Therefore, the role of the mTOR pathway in human podocytes remains to be fully evaluated.

Human induced pluripotent stem cells (hiPSCs) represent a useful model for studying both physiological and pathological conditions. However, the effects of mTOR inhibition on the process of podocyte induction from hiPSCs have not been investigated. A method of inducting podocyte differentiation from hiPSCs via nephron progenitor cells (NPCs) has been reported previously^15,16^. Therefore, in the present study, we aimed to evaluate the role of mTOR in the differentiation of podocytes from NPCs. To this end, we developed a modified differentiation protocol for the generation of podocytes from hiPSCs and, we found that the mTOR pathway is involved in podocyte differentiation. In addition, podocytes produced using our protocol can be cultured as a monolayer. Such cell monolayer could be used to evaluate permeability to relevant substances, and thereby provide a useful experimental model.

## Materials and methods

### Human iPSC culture

All the experiments involving hiPSCs were approved by the ethics committee of Kansai Medical University (Approval Number: 2020197). We obtained the written informed consent of the donors from whom hiPSCs were derived. The study was performed according to the principles of the Declaration of Helsinki, as revised in 2013, and relevant institutional guidelines. Human iPSCs (585A1, 253G1, and HiPS-RIKEN-2F) were maintained with feeder-free cells using NutriStem hPSC XF (05-100-1A, Sartorius AG, Goettingen, Germany) on plates coated with iMatrix-511 silk (892021, Matrixome, Osaka, Japan) at 37 °C in a 5% CO_2_ incubator. Single cells were prepared from hiPSC colonies (70–90% confluent) using Accutase (AT104, Innovative cell technologies, CA, USA) for subsequent passage and the induction of podocyte differentiation.

### Podocyte differentiation from hiPSCs via nephron progenitor cells

**Figure 1:**
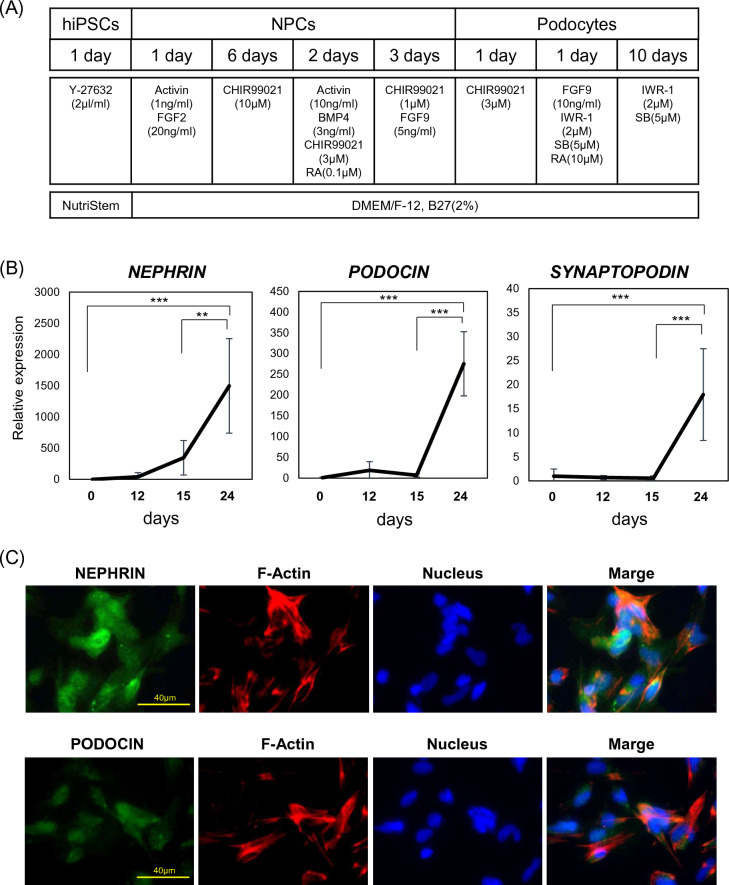

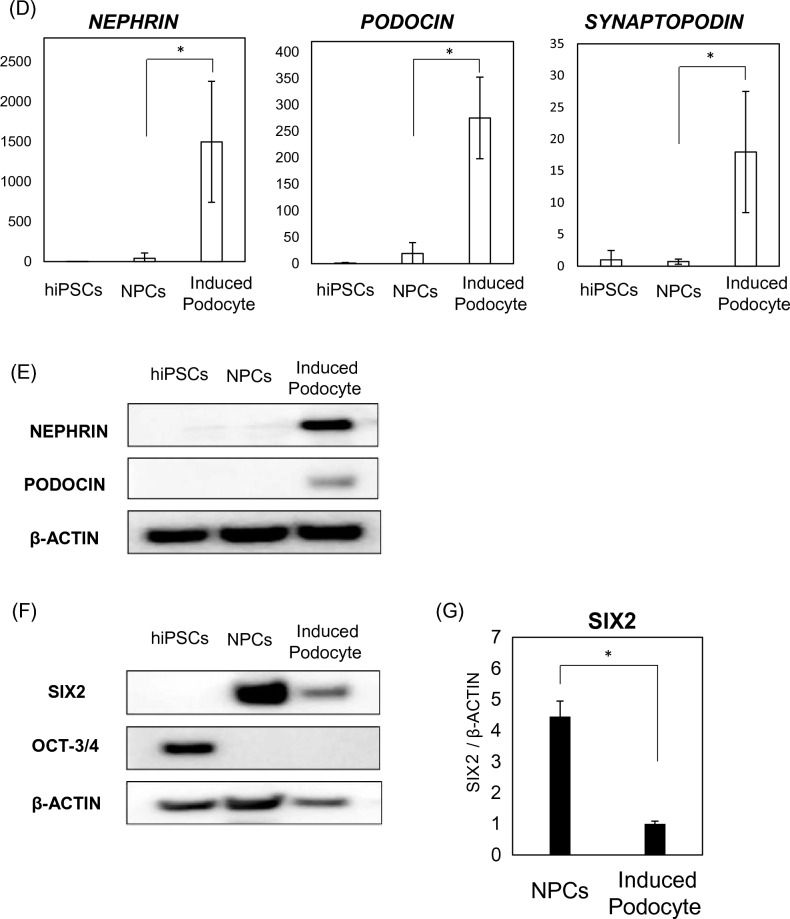
Differentiation of hiPSCs into podocyte. (**A**) Timeline and factors involved in the differentiation of hiPSCs into podocytes. (**B**) mRNA expression of podocyte-associated genes (*NEPHRIN*, *PODOCIN*, and *SYNAPTOPODIN*) during the 24 days of culture. Results are shown as the mean ± SD of 6 samples. Statistical analysis was performed using one-way ANOVA with Bonferroni’s test. **p < 0.01, ***p < 0.001. (**C**) Immunostaining for markers of podocytes (NEPHRIN and PODOCIN) and F-Actin in differentiated cells, with nuclei stained with Hoechst. (**D**) mRNA expression of podocyte-associated genes (NEPHRIN, PODOCIN, and SYNAPTOPODIN) in hiPSCs, NPCs and differentiated podocytes. Results are shown as the mean ± SD of 6 samples. Statistical analysis was performed using one-way ANOVA with Bonferroni’s test. *p < 0.05 (**E**) Protein expression of nephrin and podocin in hiPSCs, NPCs and differentiated podocytes, assessed using western blotting analysis. (**F**) Protein expression of undifferentiation stem cell marker (OCT-3/4) and nephron progenitor cell marker (SIX2) in hiPSCs, NPCs and differentiated podocytes, assessed using western blotting analysis. (**G**) Protein expression of nephron progenitor cell marker (SIX2) assessed using western blot analysis. Results are shown as the mean ± SD of 3 samples. Statistical significance was assessed using Student’s t-test. *p < 0.05.

We generated podocytes from hiPSCs by modifying a previously reported differentiation protocol^16^ (Fig. 1A). Human iPSCs were seeded at 3000 cells/well in 96 well low-cell-binding V-bottom plates, which were cultured in 200 µL NutriStem medium containing 10 μM Y27632 (FCS-10-2301-25, Focus biomolecules, PA, USA) at 37 °C for 24 h. The medium was changed to DMEM Ham’s/F12 medium (048-29775, Fujifilm, Osaka, Japan) containing 2% B27 supplement (17504044, Thermo Fisher Scientific, MA, USA), 1 ng/mL human activin A (338-AC, R&D Systems, MN, USA), and 20 ng/mL fibroblast growth factor 2 (FGF2, 064-04541, Fujifilm). After 24 h, cell aggregates were cultured for 6 days in a medium (DMEM Ham’s/F12 medium) containing 2% B27 supplement and 10 μM CHIR99021 (10-1279, Focus biomolecules) that was changed every 2 days. Subsequently, the medium was changed to one containing 10 ng/mL human activin A, 3 ng/mL human bone morphogenetic protein 4 (BMP4, PROTP12644, R&D System), 3 μM CHIR99021, and 100 nM retinoic acid (RA, 302-79-4, Fujifilm). After a further 72 h, this medium was switched to one containing 1 μM CHIR99021 and 10 ng/mL FGF9 (273-F9, R&D Systems) without medium change to induce the differentiation of NPCs.

To generate podocytes, the medium was switched to one containing 3 μM CHIR99021, and after 24 h, to one containing 2 µM IWR-1 (1127442-82-3, Fujifilm), 5 µM SB431542 (13031, Cayman Chemical, MI, USA), and 10 µM RA. After a further 24 h, the differentiated cells were cultured for 11 days in fresh medium containing 2 µM IWR-1 and 5 µM SB431542, which was replaced every 3 days. Cell sorting was not performed at all steps.

To construct the monolayer cell culture, the cell aggregates were transferred to a 50-mL centrifuge tube, washed with PBS, then dissociated using Accutase. The cells (2,000 cells/cm^2^) were then seeded onto iMatrix-511 silk-coated dishes and cultured in DMEM Ham’s/F12 medium supplemented with 10 μM Y27632 and 2% B27 supplement. Cells were collected 24 h after the treatment with DMEM Ham’s/F12 medium supplemented with Y27632 and B27 supplement.

To evaluate the involvement of the mTOR pathway in podocyte differentiation, rapamycin (R0161, LKT Laboratories, MN, USA) was administered at various times during the differentiation process and evaluated by mRNA expression using RT-PCR. In addition, S6 downstream of mTOR was inhibited using LY2584702 to further assess its involvement in the mTOR pathway.

### RT-PCR

RNA was extracted from the cells using ISOGEN II reagent (311-07361, Nippon gene, Tokyo, Japan), then a ReverTra Ace qPCR RT Master Mix (FSQ-201, Toyobo, Osaka, Japan) was used for reverse transcription. Real-time PCR was performed to quantify target mRNA expression using a Rotor-Gene Q (Qiagen) and Thunderbird SYBR qPCR Mix (QPS-201, Toyobo). The specific PCR primers used are listed (Table 1).

**Table 1:** The sequences of sense and antisense primers used for RT-PCR in this study.

Gene	Orientation	Primer sequence (5′–3′)	Gene bank association no.
*GAPDH*	Sense	TGCACCACCAACTGCTTAGC	NM001357943.2
	Antisense	GGCATGGACTGTGGTCATGAG	
*NEPHRIN*	Sense	AATCTGACAACAAGACGGAGCA	NM004646.4
	Antisense	TCGTGACATTTTCTGCCTCC	
*PODOCIN*	Sense	GGAGGCTGAAGCGCAAAGAC	AJ279254.1
	Antisense	GCCATCCTCAGGGACTCAGAAG	
*SYNAPTOPODIN*	Sense	AAATGCGTTTCTCGTTGC	BC146665.1
	Antisense	CTTCTCCGTGAGGCTAGTG	
*WT1*	Sense	AGGGTACGAGAGCGATAACCACAC	NR160306.1
	Antisense	CTCAGATGCCGACCGTACAAGA	
*MAFB*	Sense	TTGTAACCAGAATCACCCTGAGGTC	NM005461.5
	Antisense	CCAGGGTCAGGGATGGCTAA	

### Western blotting

Cell lysates were collected using 4 × Bolt LDS Sample Buffer (B0007, Thermo Fisher Scientific), then electrophoresed on a 10% SDS polyacrylamide gel and blotted onto PVDF membranes. The membranes were incubated with anti-NEPHRIN (29070, Immuno-Biological Laboratories, Gunma, Japan), anti-PODOCIN (MBS9608910, Thermo Fisher Scientific), anti-Phospho-Akt (9271, Cell Signaling Technology, MA, USA), anti-Akt (9272, Cell Signaling Technology), anti-Phospho-mTOR (2971, Cell Signaling Technology), anti-mTOR (2972, Cell Signaling Technology), anti-Phospho-p70 S6 Kinase (9205, Cell Signaling Technology), anti-p70 S6 Kinase (2708, Cell Signaling Technology), anti-Phospho-S6 Ribosomal Protein (2211, Cell Signaling Technology), S6 Ribosomal Protein (2217, Cell Signaling Technology), anti-SIX2 (80170, Cell Signaling Technology), anti-OCT3/4 (611202, BD Biosciences, NJ, USA), and anti-β actin (MAB8929, R&D Systems) primary antibodies, then further probed with anti-mouse IgG horseradish peroxidase-linked (A90-131P, Bethyl Laboratories, TX, US) secondary antibody. Specific protein bands were visualized using Pierce Western Blotting Substrate (NCI3106, Thermo Fisher Scientific).

### Flow cytometry

Cultured cells were harvested after detachment using Accutase, then incubated for 30 min at 4 °C with FITC-conjugated anti-PODOCIN antibody diluted 1:20. The cells were then centrifuged, the supernatants removed, and 500-μL aliquots of PBS containing 2% StemSure Serum Replacement (191-18375, Fujifilm) added. Data were acquired using a BD FACS Canto II flow cytometer system (BD Biosciences).

### Immunostaining

Cells were fixed using 4% paraformaldehyde, and blocked with Blocking One (03953-95, Nacalai Tesque, Kyoto, Japan) for 60 min at room temperature. Incubations were then performed at 4 °C overnight using primary anti-NEPHRIN, anti-PODOCIN antibody, and F-Actin (bs-1571R, Bioss Inc., MA, USA) antibody. Then, Alexa Fluor 488-tagged secondary antibody (ab150107, Abcam, Cambridge, UK) was applied for 30 min at room temperature, and nuclei and F-actin were stained using 10 μg/mL Hoechst 33342 (346-07951, DOJINDO Laboratories, Kumamoto, Japan) and Phalloidin-iFluor 647 Conjugate (23127, AAT Bioquest, CA, USA), respectively. The stained cells were evaluated using fluorescence microscopy (BZ-X810, Keyence, Osaka, Japan).

### Permeability assay

Podocytes differentiated from hiPSCs were seeded at 2000 cells/cm^2^ onto Transwell inserts in six-well culture plates, pore size 0.4 μm (3450, Corning, AZ, USA) coated with iMatrix-511 silk. After 24 h, DMEM Ham’s/F12 medium containing 2% B27 supplement, potassium chloride (5 mM), urea (25 mg/L), and human serum albumin (3 g/dL) were added to the lower chambers, whereas the cells were incubated in a medium lacking the latter three substances in the upper chambers. After 24 h, the media were collected from both of the chambers. The potassium concentration was measured using reagent for potassium measurement and electrode (EA09, A&T Corporation, Kanagawa, Japan). The urea nitrogen and albumin were measured using CicaLiquid-N UN reagent (77697, Kanto Chemical, Tokyo, Japan) and reagent of modified BCP method for albumin (30155001, Sekisui Medical, Tokyo, Japan), respectively, by an autoanalyzer (JCA-BM8020, JEOL Ltd., Tokyo, Japan).

### Statistical analysis

**Figure 2:**
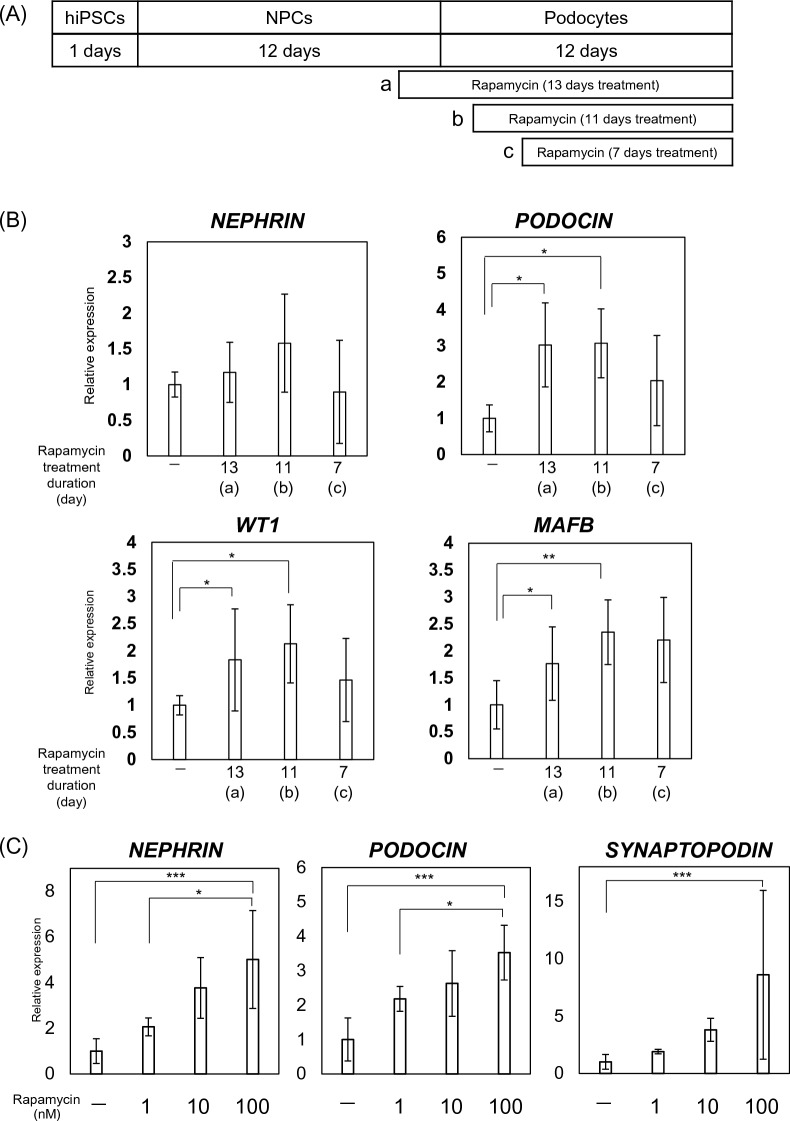

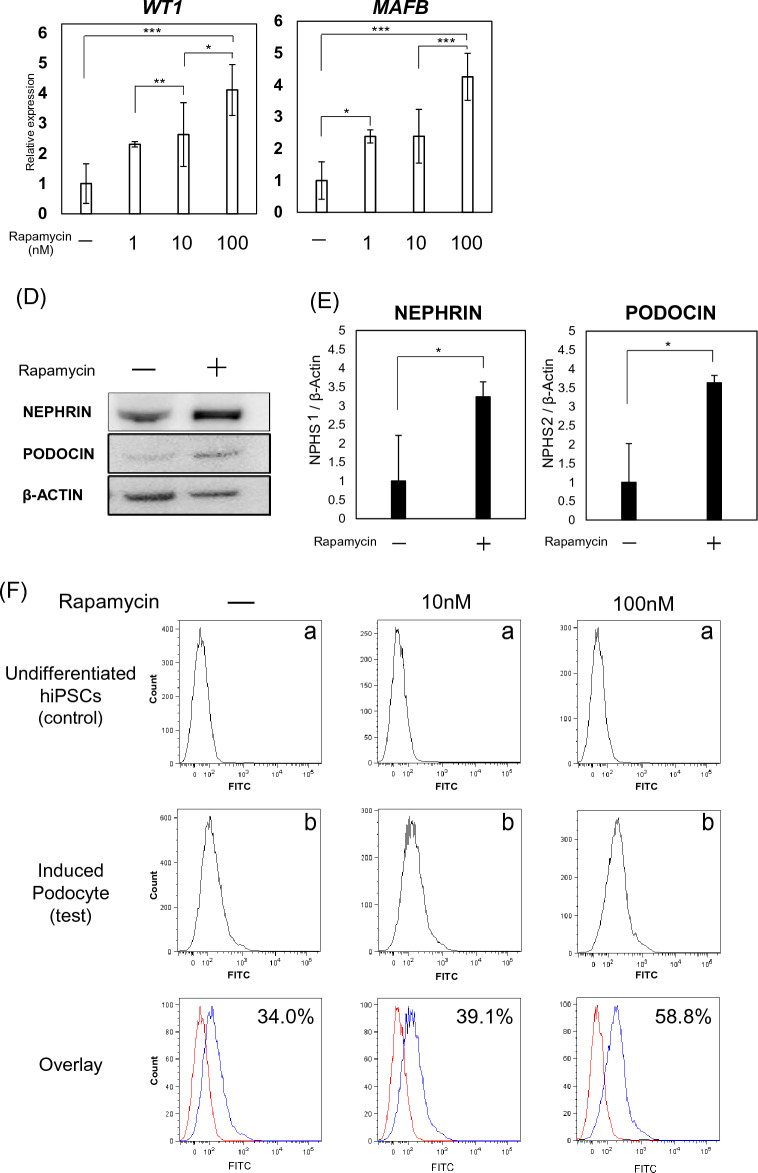
Effects of an mTOR inhibitor on podocyte differentiation. (**A**) Evaluation of the timing of rapamycin administration for protocol improvement: (a)13 days treatment, (b)11 days treatment and (c)7 days treatment. (**B**) mRNA expression of podocyte-associated genes (NEPHRIN, PODOCIN, WT1, and MAFB) in cells treated with 100 nM rapamycin at different times (a, b, c). Results are presented as mean ± SD of 6 samples. Statistical analysis was performed using one-way ANOVA with Bonferroni’s test. *p < 0.05, **p < 0.01. (**C**) mRNA expression of podocyte-associated genes (*NEPHRIN*, *PODOCIN*, *SYNAPTOPODIN*, *WT1*, and *MAFB*) in cells treated with various concentrations of rapamycin. Results are shown as the mean ± SD of 6 samples. Statistical analysis was performed using one-way ANOVA with Bonferroni’s test. *p < 0.05, **p < 0.01, ***p < 0.001. (**D**) Protein expression of nephrin and podocin in differentiated podocytes, assessed using western blotting analysis. (**E**) Protein expression of nephrin and podocin assessed using western blot analysis. Results are shown as the mean ± SD of 3 samples. Statistical significance was assessed using Student’s t-test. *p < 0.05. (**F**) Histograms for podocin-positive cells, quantified using FACS: (a) undifferentiated hiPSCs and (b) podocytes differentiated from hiPSCs.

**Figure 3:**
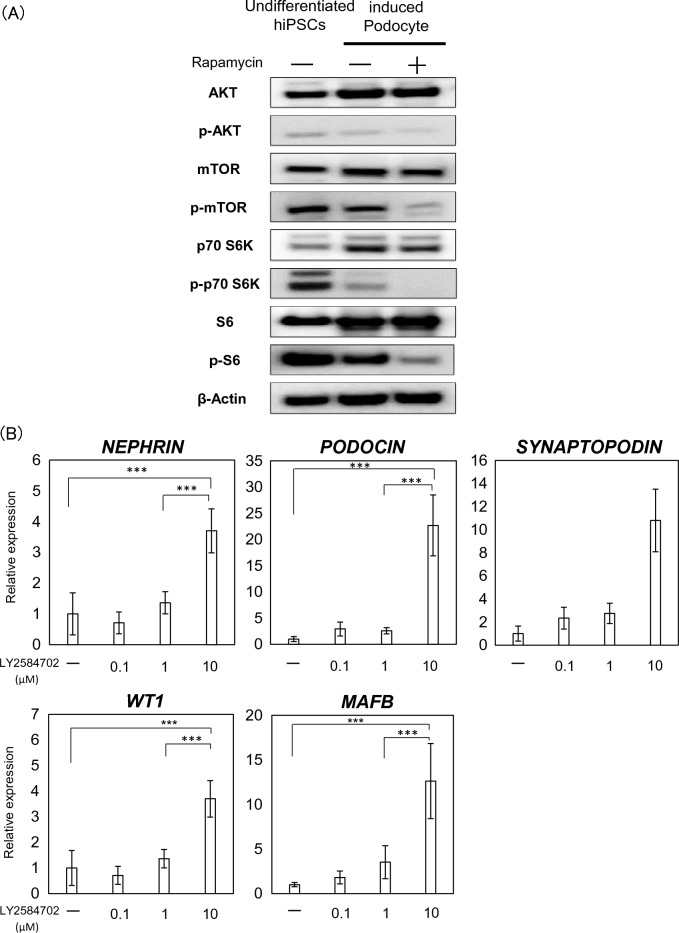
Importance of the mTOR pathway for podocyte differentiation. (**A**) Protein expression of mTOR, p-mTOR, p70 S6K, p-p70 S6K, S6, p-S6, AKT, and p-AKT, assessed using western blotting analysis. (**B**) mRNA expression of podocyte-associated genes (*NEPHRIN*, *PODOCIN*, *SYNAPTOPODIN*, *WT1*, and *MAFB*) following the addition of the S6 inhibitor LY2584702. Results are shown as the mean ± SD of 6 samples. Statistical analysis was performed using one-way ANOVA with Bonferroni’s test. ***p < 0.001.

Data are expressed as mean ± standard deviation (SD). All experiments resulted by repeating the experiment three independent times. For the results shown in Figs. 1B, 2A, and 3B, statistical analysis was performed using one-way ANOVA, followed by Bonferroni’s test; and Student’s t-tests were performed to compare the mean values of two groups for the data shown in Figs. 2C and 5B. A p-value of < 0.05 was considered to indicate statistical significance.

## Results

### Generation of podocytes from hiPSCs

We differentiated podocytes from hiPSCs using a modification of a previously reported method^16^. To improve the efficiency and stability of the podocyte differentiation, the duration and concentration of the differentiation factors were optimized (Fig. 1A). The mRNA expression of *NEPHRIN*, *PODOCIN*, and *SYNAPTOPODIN*, which are markers of podocyte differentiation, was significantly higher 24 days after differentiation than in undifferentiated hiPSCs (Fig. 1B). In contrast to the conventional differentiation protocol, the present method generated a two-dimensional monolayer (Fig. 1C). Immunostaining for podocyte markers, including F-actin, in differentiated cells revealed that almost all the cells expressed these markers (Fig. 1C). The mRNA expression of podocyte markers in differentiated cells was significantly higher than in NPCs (Fig. 1D). In addition, the protein expression levels of nephrin and podocin were significantly increased by differentiation, according to western blot analysis (Fig. 1E). Furthermore, the protein expression level of SIX2, a nephron progenitor gene, was significantly decreased in induced podocytes compared to NPCs (Fig. 1F,G). OCT-3/4 was decreased in NPCs and induced podocytes than undifferentiated hiPSCs (Fig. 1F). These results indicate that hiPSCs can be induced to differentiate to form monolayers of human podocytes.

### Effects of an mTOR inhibitor on podocyte differentiation

Rapamycin, an mTOR inhibitor, was used to evaluate the involvement of the mTOR pathway in the differentiation of podocytes from hiPSCs. Rapamycin was administered at various times during the differentiation process to assess optimal timing (Fig. 2A). Eleven days treatment with 100 nM rapamycin resulted in higher differentiation efficiency and increased mRNA expression (Fig. 2B). Cells treated with rapamycin during the differentiation process showed a concentration-dependent increase in the mRNA expression of podocyte-specific markers (Fig. 2C), suggesting that the mTOR pathway is involved in the differentiation of podocytes from hiPSCs. We also confirmed increases in the protein expression of nephrin and podocin in the cells treated with the mTOR inhibitor (100 nM, Fig. 2D,E). In addition, we evaluated the efficiency of podocyte differentiation in cells treated with and without an mTOR inhibitor (100 nM) using flow cytometric analysis (Fig. 2E). In the absence of an mTOR inhibitor, 34.0% of cells differentiated into podocytes, whereas treatment with an mTOR inhibitor dose-dependently increased the efficiency of podocyte induction, up to 58.8%.

### Importance of the mTOR signaling pathway for podocyte differentiation

**Figure 4:**
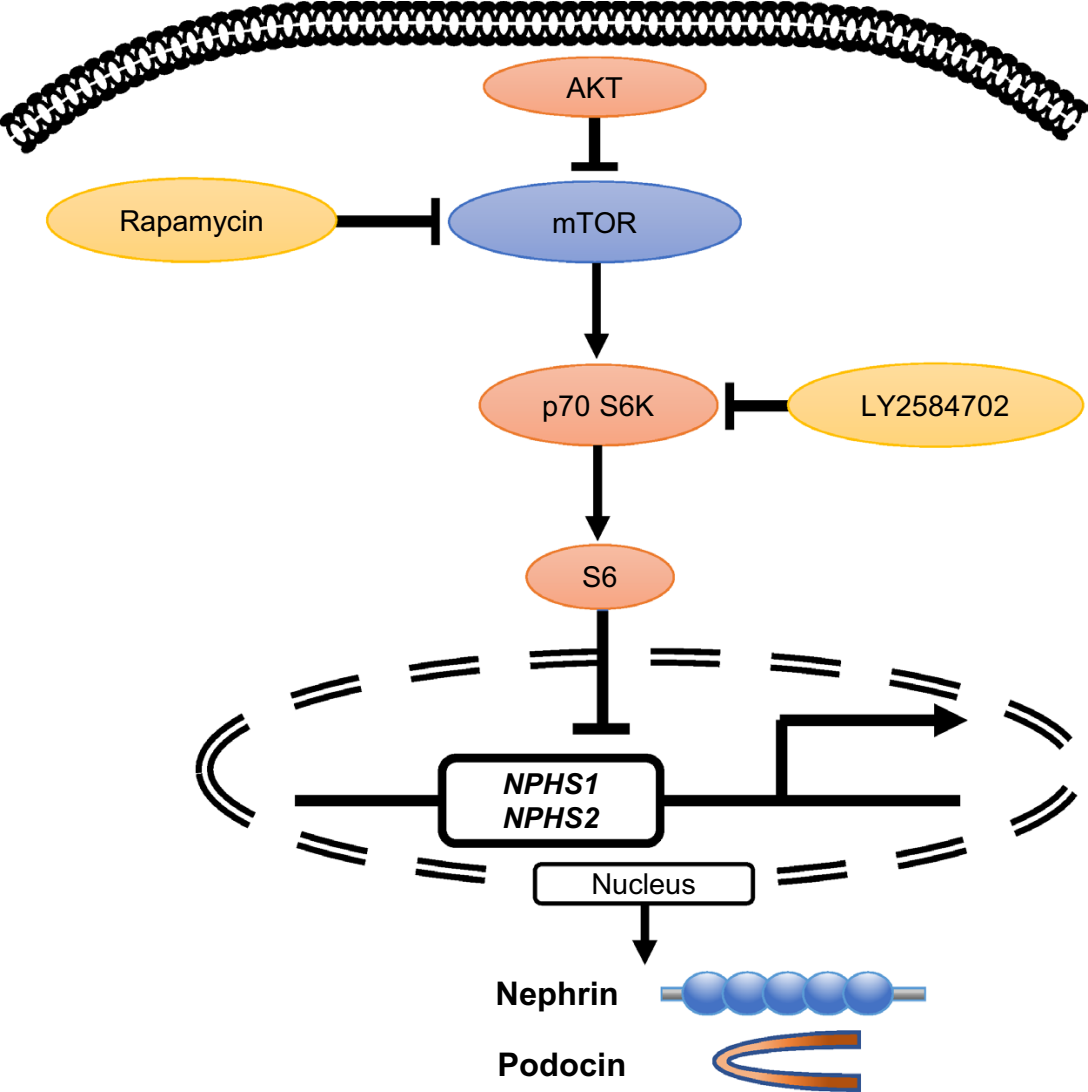
Scheme of the mTOR pathway in podocytes differentiated from hiPSCs. AKT phosphorylation attenuates mTOR phosphorylation. mTORC1 phosphorylation is inhibited by rapamycin. The mTOR pathway reduces the production of nephrin and podocin. AKT is upstream of mTOR and S6 is downstream. S6 phosphorylation is inhibited by LY2584702.

mTOR activation is regulated by a phosphorylation cascade, with AKT upstream of mTOR and S6, p70 S6K downstream. Therefore, we evaluated the phosphorylation of AKT, mTOR, S6 and p70 S6K in podocytes differentiated from hiPSCs. AKT phosphorylation did not change during podocyte induction (Fig. 3A). In addition, 100 nM rapamycin, an mTORC1 inhibitor, did not affect AKT phosphorylation, whereas the phosphorylation of both mTOR, S6 and p70 S6K was completely inhibited by rapamycin. Next, cells were treated with LY2584702, a specific S6 inhibitor for 11 days, the same duration as rapamycin. As for the mTOR inhibitor, treatment with the S6 inhibitor increased the expression of podocyte-specific markers in a concentration-dependent manner (Fig. 3B), indicating that the mTOR pathway, involving mTOR and S6 phosphorylation, is involved in the differentiation of podocytes from hiPSCs (Fig. 4).

### Biological permeability of podocytes differentiated from hiPSCs

**Figure 5:**
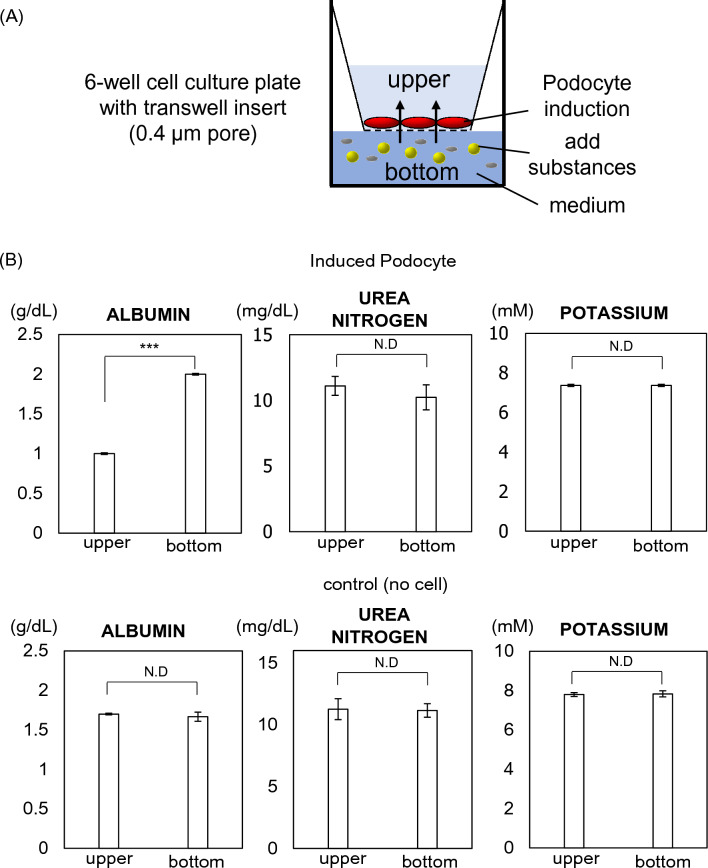
Establishment of an in vitro model of glomerular filtration. (**A**) Permeability of podocytes differentiated from hiPSCs. Differentiated podocytes were seeded into the upper chamber of six-well cell culture plates with Transwell inserts. Target substances were added to the medium in the lower chamber, and 24 h later, the media were collected from both the upper and lower chambers and evaluated. (**B**) Concentrations of albumin, urea, and potassium in the medium from each chamber. Values are shown as the mean ± SD of 3 samples. Statistical significance was assessed using Student’s t-test. ***p < 0.001.

Glomerular filtration is an important facet of kidney function and podocytes are key determinants of the permeability of nephrons. Therefore, we generated an in vitro glomerular filtration model that consisted of a two-dimensional monolayer of podocytes differentiated from hiPSCs (Fig. 5A). Urea and potassium are used as clinical markers for the evaluation of kidney function and are not efficiently filtered from the plasma in patients with renal dysfunction, whereas albumin is a plasma protein that is important for the maintenance of effective circulating plasma volume and is not normally filtered. Urea, potassium, and albumin were added to the medium of the lower wells at concentrations that are similar to those in the plasma of healthy humans (final concentrations: urea, 25 mg/dL; potassium, 5.0 mM; albumin 3.0 g/dL). Conditioned media were collected from both the upper and lower chambers after 24 h and the concentrations of each solute were measured. The permeabilities to urea and potassium were nearly 100% and there was no difference from the one without podocyte induced from hiPSCs, whereas the permeability to albumin was low, because concentration of upper chamber was significantly lower in the podocyte-conditioned medium (Fig. 5B).

## Discussion

Podocytes play pivotal roles in the maintenance of the glomerular filtration barrier and structural integrity. Consequently, podocyte deterioration causes defects in filtration and proteinuria in patients with kidney diseases. Because human podocytes are difficult to obtain for in vitro experiments, the physiology and pathophysiology of podocytes remain unclear. In the present study, we developed a protocol for the differentiation of human podocytes from hiPSCs by modifying a previous protocol. In addition, we have developed a model of glomerular filtration using a monolayer of podocytes. Monolayers are more useful for the evaluation of pathophysiology in podocytes than conventional cell aggregates, as previously reported by Musah et al.^15^. In the present study, immunostaining for podocyte markers revealed that these markers were not expressed in permeabilized cells, but were expressed in non-permeabilized cells. This suggests that nephrin and podocin, which comprise the glomerular filtration slits, are present in the cell membranes of the differentiated podocytes. This showed that the monolayer was permeable to urea and potassium, whereas its permeability to albumin was only 50%. This in vitro experimental model provides us with a tool with which to elucidate the mechanism of glomerular filtration and evaluate pathological changes.

There are several advantages of the podocyte differentiation method developed in the present study. Firstly, the new method does not require cell sorting; therefore, we consider that the cell damage associated would be lower than that associated with the previous protocol. Secondly, monolayers are more useful for the evaluation of and glomerular filtration and its pathology, as discussed above. Finally, there have been no reports using mTOR inhibitors in the protocol to differentiation of podocytes from hiPSCs, and we have shown that using mTOR inhibitors increases the efficiency of induction to podocytes. Here, we showed a detailed comparison with the previous differential protocol (Fig. S1). We considered that our differentiation method was not a substantial improvement over the earlier methods. On the other hand, a previous method^16^ did not involve NPCs. Our method can be compared between NPCs and differentiated podocytes. In addition, another protocol^15^ did not perform a monolayer culture. The aim of this study was to evaluate the role of mTOR in the differentiation of podocytes from NPCs. We believe that our differentiation method and results achieved this aim. However, there is a limitation of the new method: the induction rate of differentiated podocytes generated may be lower than that generated using the conventional method with cell sorting. Monolayer cell cultures generally comprise fewer cells than cell aggregates. Therefore, when pure podocytes are needed for regenerative medicine or basic research, it may be preferable to use a protocol involving conventional cell aggregates. In addition, we have demonstrated the involvement of the mTOR pathway in podocyte differentiation, but the role of mTOR in mature podocytes in adults is unclear. Thus, the role of mTOR in well-differentiated podocytes should be investigated to determine whether mTOR inhibition might be of therapeutic utility under pathophysiological conditions. Furthermore, the ability to model glomerular function using differentiated podocytes may be limited, because filtration involves vascular endothelial cells and glomerular basement membrane, as well as the podocytes used in this model.

We have also shown that the mTOR pathway is involved in the differentiation of podocytes from hiPSCs. mTOR has key roles in a number of cell functions, including growth, differentiation, and metabolism^7^. mTOR forms two complexes, mTORC1 and mTORC2, from several molecules, including mammalian lethal with SEC13 protein 8 and regulatory-associated protein of mTOR. mTORC1 is inhibited by rapamycin, which was used in the present study, and the inhibition of mTORC1 promotes protein synthesis, suppresses protein degradation, and consequently promotes cell growth. The activation of mTORC1 results in the phosphorylation of two downstream targets, ribosomal S6 kinase and eukaryotic translation initiation factor 4E-binding protein, which stimulate ribosomal biogenesis and protein translation to increase cell mass^17,18^. However, the role of the mTOR pathway in human podocytes remains controversial. Although the mTOR pathway has been reported to play roles in the physiology and pathophysiology of podocytes, the molecular mechanisms involved have not been fully investigated, because of the lack of availability of cultured human podocytes. The study of the differentiated human podocytes showed that mTOR is involved in podocyte differentiation and that the inhibition of mTOR promotes podocyte differentiation, which may lead to further efficiency in the methods available to induce podocyte differentiation. Future studies should evaluate the involvement of the mTOR pathway in pathological conditions in human podocytes and may lead to the development of novel therapies for nephropathy that involve mTOR inhibition. Furthermore, we have not evaluated its role in the distinctive microstructures of podocytes, such as foot processes, and further studies are also needed in this area, because microstructural defects are important in kidney diseases, including in minimal change nephrotic syndrome and diabetic nephropathy.

In conclusion, we have established a modified protocol for the differentiation of podocytes from hiPSCs without the need for cell sorting and with minimal damage. The differentiated podocytes generated may be useful for the analysis of pathology and the development of in vitro glomerular models. We have also shown that the mTOR pathway is involved in podocyte differentiation and may also be involved in the podocyte deterioration that characterizes kidney diseases. Thus, the monolayer podocyte differentiation protocol, using an mTOR inhibitor, provides a novel model for the study of physiology and pathophysiology.

### Supplementary Information

Supplementary Figures.

## Data Availability

The data that support the findings of this study are available from the corresponding author upon reasonable request.
