# Supplementary material for: Efficient protocol for the differentiation of kidney podocytes from induced pluripotent stem cells, involving the inhibition of mTOR
Source: Sci Rep. 2023 Nov 16;13:20010. doi: 10.1038/s41598-023-47087-8 (PMC10654390; doi:10.1038/s41598-023-47087-8)
Supplement: Supplementary file 1 — Supplementary Figures. [file 41598_2023_47087_MOESM1_ESM.docx]

Supplementary Materials for

**Efficient protocol for the differentiation of kidney podocytes from induced pluripotent stem cells, involving the inhibition of mTOR**

Masahiro Yasuda^1,2^, Tadashi Kato^1,3^, Mai Okano^1,2^, Hiromi Yamashita^1^, Yoshikazu Matsuoka^1^, Yasumasa Shirouzu^1^, Tatsuya Fujioka^1^, Fumiyuki Hattori^1^, Shoji Tsuji^2^, Kazunari Kaneko^2^, Hirofumi Hitomi^1^

^1^ Department of iPS Stem Cell Regenerative Medicine, Kansai Medical University, Osaka, Japan

^2^ Department of Pediatrics, Kansai Medical University, Osaka, Japan.

^3^ Division of Nephrology, Department of Medicine, Showa University School of Medicine, Tokyo, Japan

Figure S1. Differentiation methods for generating podocytes from human induced pluripotent stem cells

Figure S2. Original blots of Figures

**Supplemental Figures**

**
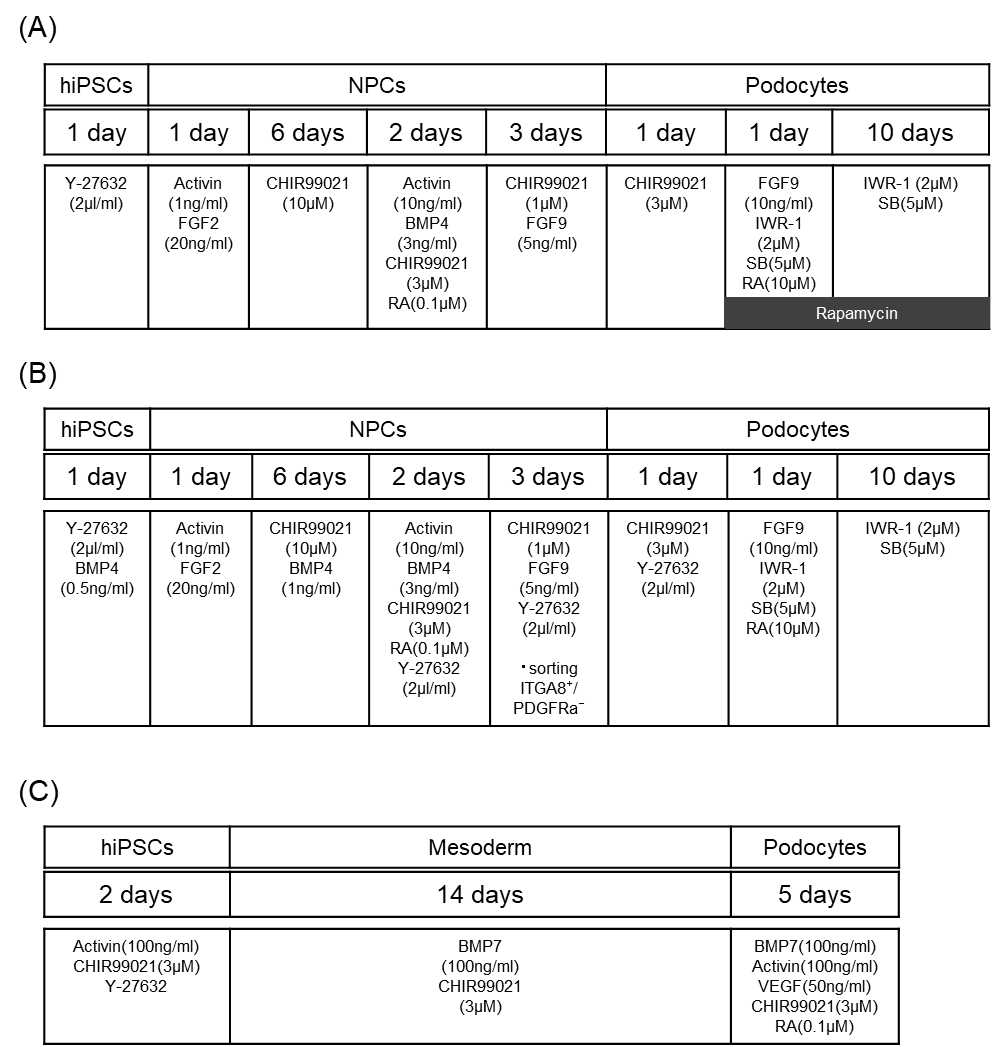
**

**Figure S1. Differentiation methods for generating podocytes from human induced pluripotent stem cells**

The differentiation of podocytes from hiPSCs was performed by a protocol of the present study or previously reported protocols. **(A)** present study. **(B)** previously reported protocol of Ref 16. **(C)** previously reported protocol of Ref 15.


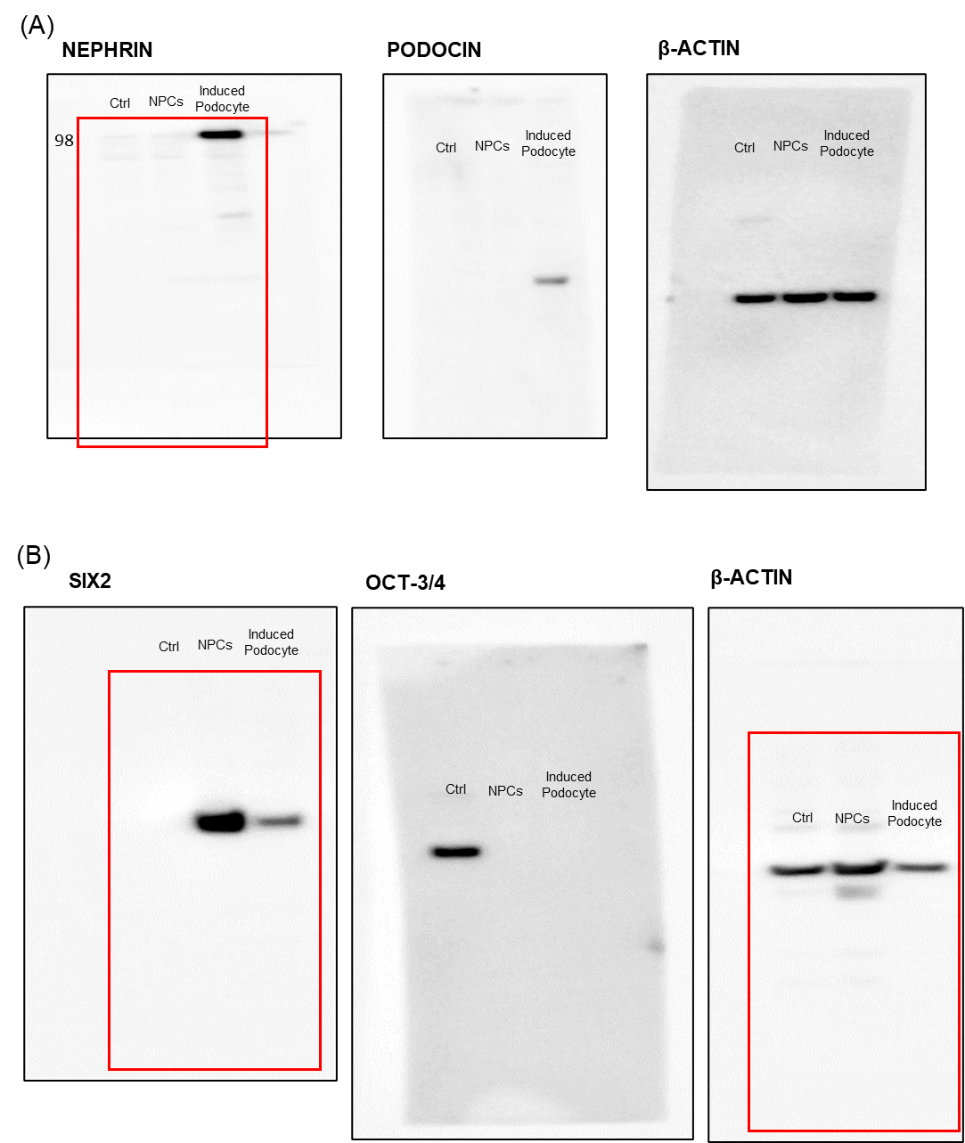


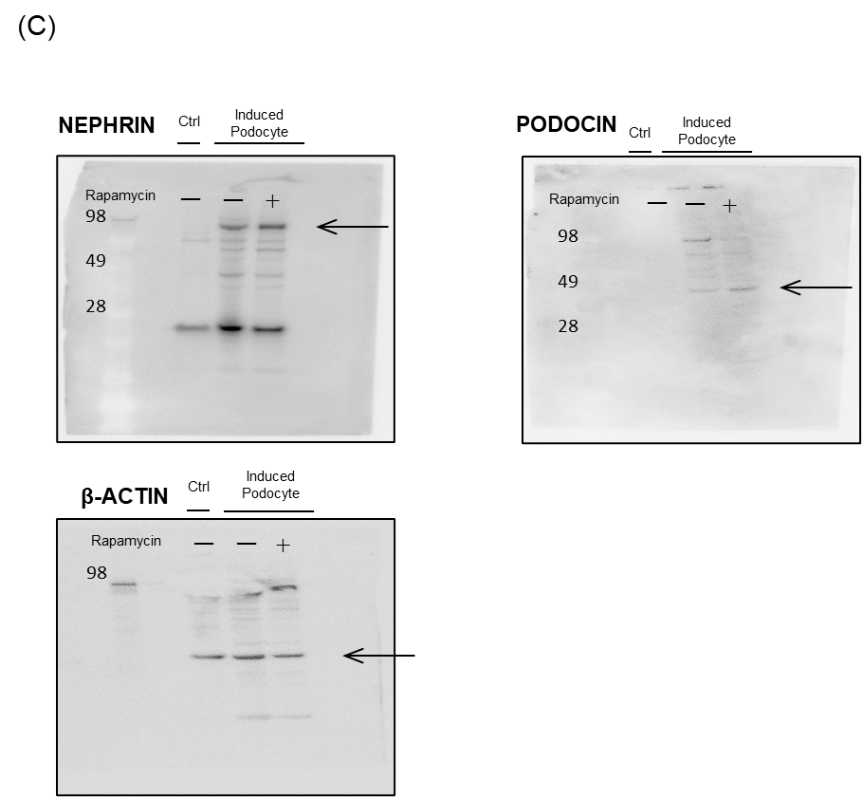


**
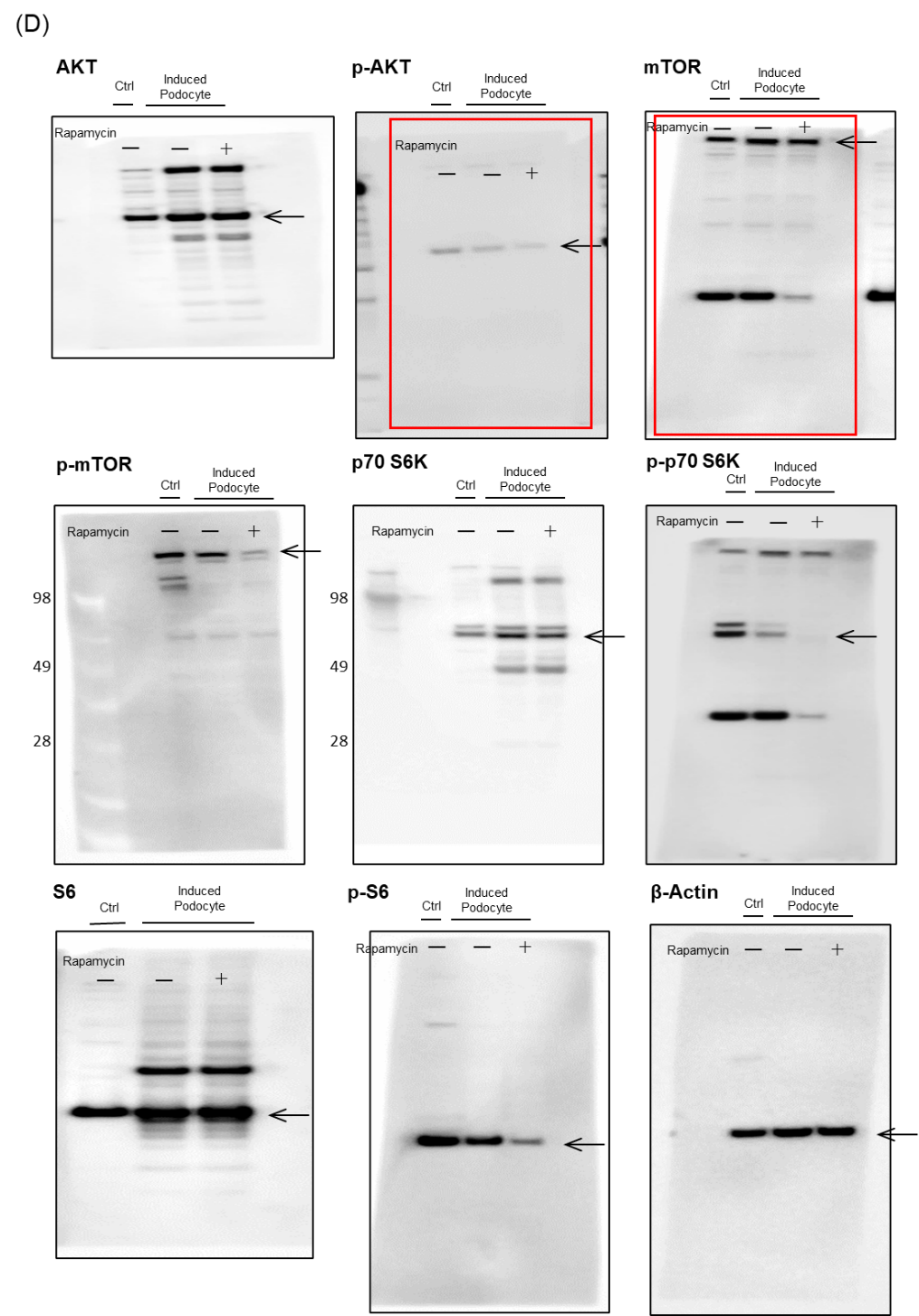
**

**Figure S2. Original blots of Figures**

**(A)** The original images for cropped gel are presented in Figure 1E. **(B)** The original images for cropped gel are presented in Figure 1F. **(C)** The original images for cropped gel are presented in Figure 2D. **(D)** The original images for cropped gel are presented in Figure 3A.
